# The links between the amount of antipsychotic medication prescribed at GP practice level, local demographic factors and medication selection

**DOI:** 10.1192/bjo.2021.458

**Published:** 2021-06-18

**Authors:** Adrian Heald, Mike Stedman, Sanam Farman, Mark Davies, Roger Gadsby, David Taylor

**Affiliations:** 1 University of Manchester, Salford Royal Hospital; 2 Res Consortium; Chaw Khine, Kings Mill Hospital; 3 Mersey Deanery Psychiatry Rotation; 4 Res Consortium; 5 University of Warwick; 6 South London and Maudsley NHS Foundation Trust

## Abstract

**Aims:**

To examine the factors that relate to antipsychotic prescribing in general practices across England and how these relate to cost changes in recent years.

**Background:**

Antipsychotic medications are the first-line pharmacological intervention for severe mental illnesses(SMI) such as schizophrenia and other psychoses, while also being used to relieve distress and treat neuropsychiatric symptoms in dementia.

Since 2014 many antipsychotic agents have moved to generic provision. In 2017_18 supplies of certain generic agents were affected by substantial price increases.

**Method:**

The study examined over time the prescribing volume and prices paid for antipsychotic medication by agent in primary care and considered if price change affected agent selection by prescribers.

The NHS in England/Wales publishes each month the prescribing in general practice by BNF code. This was aggregated for the year 2018_19 using Defined Daily doses (DDD) as published by the World Health Organisation Annual Therapeutic Classification (WHO/ATC) and analysed by delivery method and dose level. Cost of each agent year-on-year was determined.

Monthly prescribing in primary care was consolidated over 5 years (2013-2018) and DDD amount from WHO/ATC for each agent was used to convert the amount to total DDD/practice.

**Result:**

Description

In 2018_19 there were 10,360,865 prescriptions containing 136 million DDD with costs of £110 million at an average cost of £0.81/DDD issued in primary care. We included 5,750 GP Practices with practice population >3000 and with >30 people on their SMI register.

Effect of price

In 2017_18 there was a sharp increase in overall prices and they had not reduced to expected levels by the end of the 2018_19 evaluation year. There was a gradual increase in antipsychotic prescribing over 2013-2019 which was not perturbed by the increase in drug price in 2017/18.

Regression

Demographic factors

The strongest positive relation to increased prescribing of antipsychotics came from higher social disadvantage, higher population density(urban), and comorbidities e.g. chronic obstructive pulmonary disease(COPD). Higher %younger and %older populations, northerliness and non-white (Black and Minority Ethnic (BME)) ethnicity were all independently associated with less antipsychotic prescribing.

Prescribing Factors

Higher DDD/general practice population was linked with higher %injectable, higher %liquid, higher doses/prescription and higher %zuclopenthixol. Less DDD/population was linked with general practices using higher %risperidone and higher spending/dose of antipsychotic.

**Conclusion:**

Higher levels of antipsychotic prescribing are driven by social factors/comorbidities. The link with depot medication prescriptions, alludes to the way that antipsychotics can induce receptor supersensitivity with consequent dose escalation.

